# Microneedle Sensors for Point‐of‐Care Diagnostics

**DOI:** 10.1002/advs.202306560

**Published:** 2024-01-15

**Authors:** Yubing Hu, Eleni Chatzilakou, Zhisheng Pan, Giovanni Traverso, Ali K. Yetisen

**Affiliations:** ^1^ Department of Chemical Engineering Imperial College London London SW7 2AZ UK; ^2^ Department of Mechanical Engineering Massachusetts Institute of Technology Cambridge MA 02139 USA

**Keywords:** biosensors, diagnosis, microneedles, point‐of‐care, transdermal monitoring

## Abstract

Point‐of‐care (POC) has the capacity to support low‐cost, accurate and real‐time actionable diagnostic data. Microneedle sensors have received considerable attention as an emerging technique to evolve blood‐based diagnostics owing to their direct and painless access to a rich source of biomarkers from interstitial fluid. This review systematically summarizes the recent innovations in microneedle sensors with a particular focus on their utility in POC diagnostics and personalized medicine. The integration of various sensing techniques, mostly electrochemical and optical sensing, has been established in diverse architectures of “lab‐on‐a‐microneedle” platforms. Microneedle sensors with tailored geometries, mechanical flexibility, and biocompatibility are constructed with a variety of materials and fabrication methods. Microneedles categorized into four types: metals, inorganics, polymers, and hydrogels, have been elaborated with state‐of‐the‐art bioengineering strategies for minimally invasive, continuous, and multiplexed sensing. Microneedle sensors have been employed to detect a wide range of biomarkers from electrolytes, metabolites, polysaccharides, nucleic acids, proteins to drugs. Insightful perspectives are outlined from biofluid, microneedles, biosensors, POC devices, and theragnostic instruments, which depict a bright future of the upcoming personalized and intelligent health management.

## Graphical abstract

Microneedle sensors with diverse geometries, materials and fabrication methods have emerged as a minimally invasive analytical platform to detect a wide range of biomarkers in interstitial fluid. The “lab‐on‐a‐microneedle” platform has been elaborated with various sensing techniques to achieve point‐of‐care diagnostics in a conformable, low‐cost, accurate, and real‐time manner.

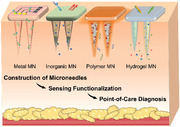

## 1 Introduction

Healthcare diagnostics, as the first step to getting effective treatment, has attracted tremendous endeavors for promoting invasive blood‐centered diagnosis by benchtop analytical instruments in hospital settings to noninvasive biofluid diagnosis by portable and wearable devices in point‐of‐care (POC) settings.^[^ ^1^, ^2^ ^]^ POC testing offers rapid test results at or near the patient's site, which facilitates decentralized diagnosis and personalized medicine. The global market of POC diagnostics is growing rapidly, which was estimated to be worth $45.4 billion in 2022 and is poised to reach $75.5 billion by 2027.^[^ ^3^ ^]^ The development of robust, rapid, and cost‐effective POC diagnostic devices to supplement standard laboratory‐based diagnostic tools and reduce hospital burden has been a focus of research.^[^ ^4^ ^]^ Blood serum and plasma have been long known as the gold standard for definitive diagnosis, while blood sampling by invasive finger pricking causes pain, bruising, and possible infection. Minimally‐ or non‐invasive diagnosis of easy‐accessible body fluids like interstitial fluid (ISF), urine, and saliva is expected to advance blood‐based clinical care. Among these biofluids, ISF is formed by capillary filtration of blood and has been recognized as the most potent alternative to blood due to its similar composition to plasma and most abundant component (75‒80%) of extracellular fluid.^[^ ^5^ ^]^ Moreover, ISF with the surrounding cells and tissues can reach continuous monitoring of wide‐ranging molecular, cellular, histologic, and physiologic biomarkers.^[^ ^6^ ^]^ Although ISF mainly exists in the easily approached skin, the establishment of a simple and effective ISF sampling method remains challenging for POC diagnosis by users without medical expertise.^[^ ^7^ ^]^

Despite their effectiveness for ISF collection, the conventional ISF sampling techniques are undermined for POC diagnosis for certain limitations such as insufficient volume (1‐10 µL) and filtration.^[^ ^8^ ^]^ For example, biopsy^[^ ^9^ ^]^ sampling causes great pain, and even leads to permanent skin damage; suction blisters^[^ ^10^ ^]^ last for hours and induces lasting skin wound; reverse iontophoresis^[^ ^11^, ^12^ ^]^ suffers from the limitation to small molecules and frequent calibrations; sonophoresis^[^ ^13^ ^]^ requires a professional ultrasound operation and shows inconsistent skin permeability, open‐flow microperfusion^[^ ^14^ ^]^ and microdialysis^[^ ^15^, ^16^ ^]^ samplings by invasive implantation of tubing and membrane are tedious and complicated. In comparison to these techniques, microneedles have emerged as an attractive safe and painless ISF sampling tool.^[^ ^17^ ^]^ The micrometer‐scale needles can puncture micro‐holes in the stratum corneum (SC), unlikely to reach the blood capillaries and heal within minutes, hence preventing bacterial infection.^[^ ^18^ ^]^ To date, the most prevalent application of microneedles has focused on the enhanced transdermal delivery of drugs and vaccines.^[^ ^19^ ^]^ Further advanced applications in skincare cosmetics,^[^ ^20^ ^]^ hair regrowth,^[^ ^21^ ^]^ sports analytics,^[^ ^22^ ^]^ antibacterial therapeutics,^[^ ^23^ ^]^ cancer detection^[^ ^24^, ^25^ ^]^ have been explored by microneedles. Since the first report on silicon microneedle‐based glucose sensing in 2000,^[^ ^26^ ^]^ the interest in microneedles to bypass the SC barrier and acquire transdermal monitoring of biomarkers has rapidly increased. Exploiting microneedle patch sensors is beneficial for routine POC diagnostics due to its painless, user‐friendly, and low‐cost advantages. The current microneedle‐based diagnostic devices are dominated by microneedle arrays functionalized by biochemical sensors and assembled on a miniature patch, as revealed by few reviews.^[^ ^27^, ^28^, ^29^, ^30^ ^]^ These reviews either follow the traditional classification of microneedles or the classic category of sensors distinguished by biomarkers, thus cannot give a systematic overview of current microneedle sensors to speed up the clinical practice.

Recently, the integration, miniaturization, and functionalization of micro‐sensing systems have been accelerated due to continuous innovations in various fields such as micro/nano‐fabrication technology,^[^ ^31^, ^32^ ^]^ materials science,^[^ ^33^, ^34^ ^]^ digital technology,^[^ ^35^, ^36^ ^]^ biosensing platforms,^[^ ^37^, ^38^ ^]^ and flexible electronics.^[^ ^39^, ^40^ ^]^ These advancements represent a significant milestone in microneedle‐based transdermal sensors and hold immense potential for revolutionizing the healthcare field toward minimally invasive, real‐time, and long‐term POC diagnostics.^[^ ^30^ ^]^ Nevertheless, the successful integration of microneedles into biomedical devices necessitates the achievement of exceptional stability, accuracy, and biocompatibility over an extended period.^[^ ^41^ ^]^ Additionally, the materials used in microneedles must strike a delicate balance between reliable penetration performance and gentle interaction with the skin. This review provides a vision of “lab‐on‐a‐microneedle” technology and a clear framework of microneedle sensors from materials perspectives that can keep pace with rapidly evolving microneedle research and identify the requirements for future development in POC diagnostics. To begin, we provide a brief introduction to the concept, design strategies, and working principles of microneedle sensors. We then compare various microneedle fabrication techniques and present the accessible biological matrices (e.g., blood, interstitial and lymphatic fluid, cells and tissues) for healthcare monitoring. Most importantly, we delve into a comprehensive study of state‐of‐the‐art microneedle sensors after analyzing the pros and cons of various microneedle materials classified by four categories: metals, inorganics, polymers, and hydrogels. Microneedle sensors for the detection of biomarkers ranging from electrolytes (e.g., H^+^, Na^+^, K^+^, and Ca^2+^), metabolites (e.g., glucose, lactate, ascorbic acid, dopamine), polysaccharides (e.g., endotoxin), nucleic acids (DNA and miRNA), proteins (e.g., cytokine, tyrosinase, thrombin), and drugs (e.g., apomorphine, tobramycin) are presented. Lastly, we outline the future development trajectory of microneedle sensing systems while highlighting the challenges and risks involved in their applications in POC diagnostics.

## 2 The Fundamentals of Microneedle Sensors

### 2.1 Design Principles of Microneedle Sensors

**Figure 1:**
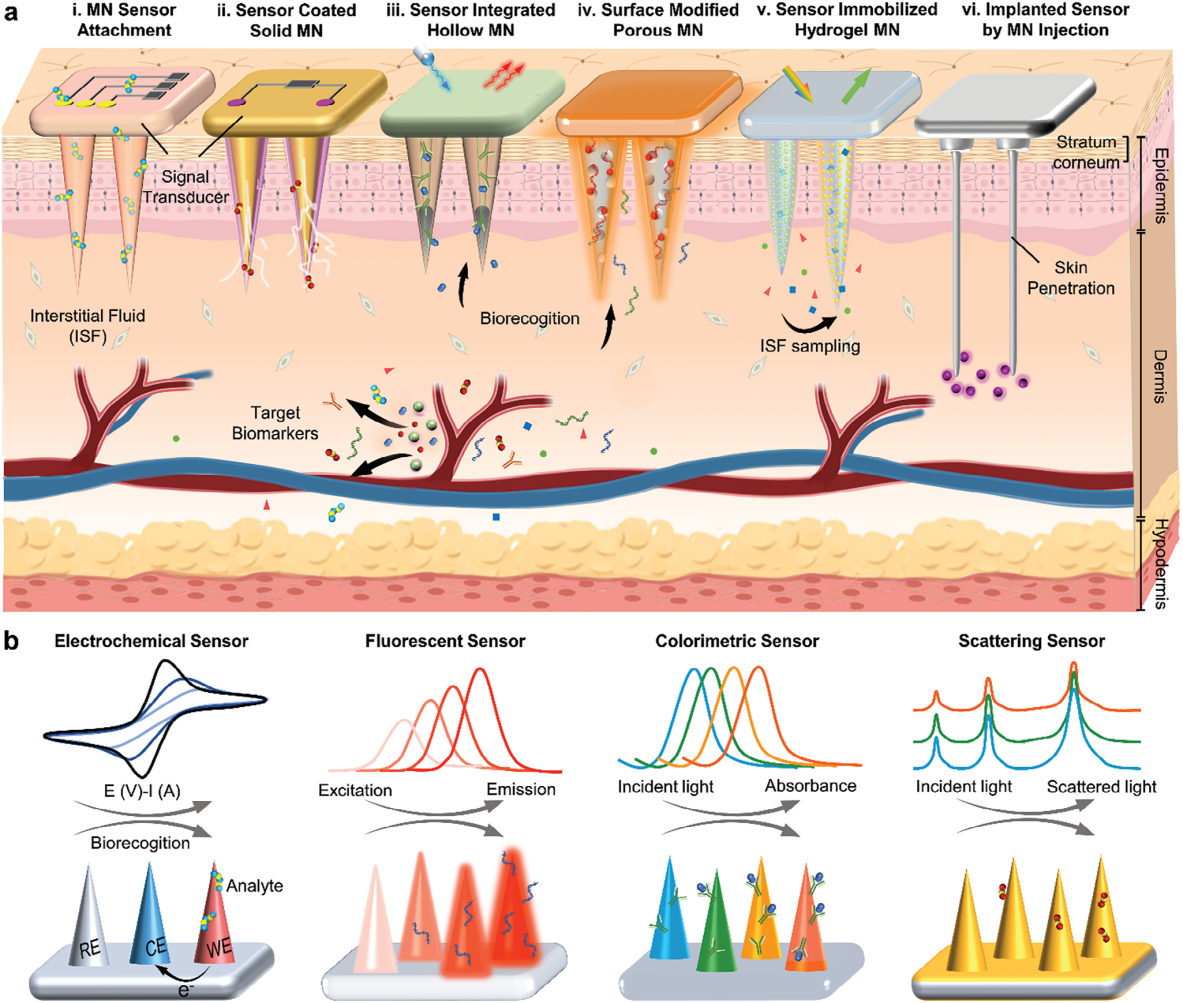
Design and working principles of microneedle sensors. a) Schematic representation of the skin penetration and biomedical diagnosis by microneedle (MN) sensors constructed by various “lab‐on‐a‐microneedle” technologies. b) Microneedle sensors based on different sensing principles.

Microneedles have been observed in various natural systems, such as the mouthparts of female mosquitoes with fork‐like tips and micro/nano‐saw teeth.^[^ ^42^ ^]^ These natural microneedles are not limited to mosquitoes but are also found in other organisms, including honeybees,^[^ ^43^ ^]^ mantis,^[^ ^44^ ^]^ rear‐fanged snakes,^[^ ^45^ ^]^ highlighting their widespread existence in nature. Microneedles are designed to penetrate the epidermis, particularly the SC and essential epidermis. As a minimally‐invasive alternative to conventional intrusive needles, microneedles are originally employed to administer medications and vaccinations transdermally.^[^ ^46^ ^]^ The skin consists of three layers: the epidermis, dermis, and hypodermis (**Figure** **1**a). Among them, the epidermis is the outermost layer without blood vessels and contains the lowest amount of ISF (15‐35% by mass). In contrast, the dermis has a higher concentration of ISF, making it an excellent choice for microneedle sensing. The tiny microneedles, manufactured in solid, hollow, coated, and dissolved configurations,^[^ ^47^ ^]^ almost have no contact with the nerve terminals and thus realize the painless and comfortable sampling. Solid microneedles, known for their mechanical stability and modification potential, create microincisions in the skin to collect body fluids through direct penetration.^[^ ^48^ ^]^ In the realm of transdermal sensing, hydrogel microneedles, a unique variant of solid microneedles that can swell post‐penetration, serve the same purpose, loading selective materials onto their surface and capturing analytes. They offer adjustable stiffness for different skin types, excellent biocompatibility, and cost‐effective, straightforward mass production.^[^ ^49^ ^]^ Hollow microneedles use capillary force to extract skin fluids and can be accurately fabricated with open microfluidic channels or conductive materials for electrochemical sensing.^[^ ^48^, ^49^ ^]^ A key issue is the potential blockage of channels by trapped skin tissue.^[^ ^50^ ^]^ Coated microneedles incorporate a surface‐coated core for drug and biomarker monitoring. Dissolvable microneedles, which can also be used for ISF extraction, offer versatile applications in drug delivery.^[^ ^48^, ^51^ ^]^ Microneedle design parameters, including length, width, thickness, tip angle, and needle distribution, affect pain perception and successful penetration. Longer needles (>1100 µm) can significantly increase pain during insertion, with factors like pressure amount, fluid type, and insertion mechanism playing a role.^[^ ^50^, ^52^ ^]^ Furthermore, microneedle aspect ratio is a critical geometric consideration. It can be defined in different ways (length over the tip diameter of the needle, length over the width of the microneedle, or base over the tip diameter) but it's essential to be kept below 2.0 to prevent needle overlap.^[^ ^52^, ^53^ ^]^ Miniaturizing hypodermic needles to micron size is a promising approach for minimally invasive, less painful, and low infection risk biofluid extraction.^[^ ^50^ ^]^

Microneedle platforms aim to combine biomolecule analysis within the device by integrating sensing technologies. They have been recently utilized as a powerful wearable sensor platform to detect clinically significant analytes and to monitor biomarkers or medicines transdermally for regular POC health monitoring.^[^ ^54^, ^55^ ^]^ The “lab‐on‐a‐microneedle” technology using engineered functional materials in micro‐ and nano‐structured microneedles is proposed as a cohesive way to translate materials science and biotechnology in microneedles.^[^ ^56^ ^]^ According to the integration strategies of sensors in microneedles, the architecture of “lab‐on‐a‐microneedle” technology can be classified into six categories: i) attachment of a sensor to the microneedle base. ii) coating of sensors in microneedle surface. iii) integration of sensors in hollow microneedles. iv) sensing modification of porous microneedle. v) immobilization of sensors in microneedle matrix. vi) injection of sensors by microneedle. vi) injection of sensors by microneedle.

The sensing technologies can be divided into four major categories based on the transduction techniques they employ classified as mass, magnetic, electrochemical, and optical (Figure 1b). After decades of development, microneedle‐based biosensors are mainly coupled with electrochemical and optical detection. Depending on the observed parameter, transduction elements in electrochemical‐based biosensors can be classified as amperometric, potentiometric, or impedimetric electrodes. Electrochemical biosensors offer immense possibilities as next‐generation detection systems due to their high sensitivity, low cost, ease of operation, and miniaturization, especially when integrated with suitable microneedle‐based probes that generate electron transfer.^[^ ^58^ ^]^ Owing to the electrical conductivity or electrochemical reactivity of the commonly deployed microneedle materials, continuous monitoring of analytes including neurotransmitters and pathogenic microbes, could enable real‐time detection of physiological processes.^[^ ^59^ ^]^ However, broad‐spectrum detection of various biomarkers is an obvious drawback of electrochemical detection. Optical‐based biosensors have become prevalent because they combine many different types of techniques, such as absorption, emission (including fluorescence, chemiluminescence, bioluminescence, and phosphorescence), reflection or refraction, and scattering, e.g., surface‐enhanced Raman spectroscopy (SERS), with high sensitivity, specificity, affordability, and small size. Optical transducers also provide the potential for label‐free, real‐time detection. When microneedles are used as fluorescent probes, they generate a fluorescent signal upon interaction with the target analyte providing a quantitative output.^[^ ^60^, ^61^ ^]^ Since in many cases it is required that the microneedles absorb the target marker to enable the interaction with the entrapped fluorophores in the main microneedle matrix, the swelling properties of certain types of microneedles, namely hydrogels, can be proven very advantageous. Fluorescence detection has the advantages of intuitive signal generation and convenient reading. However, because the fluorescence signal is greatly affected by the penetration depth and the surrounding environment of the tissue, it is challenging to quantitatively analyze the markers. Colorimetry measures the concentration of an analyte using a specific reaction that results in a visible color shift, due to alteration of the lattice structure of the microneedles once the target analyte is absorbed.^[^ ^62^ ^]^ Although it has several drawbacks, such as sample reliance and poor bioreceptor immobilization, it is commonly employed in commercial biosensors due to the offered specificity, portability, and low cost.^[^ ^63^ ^]^ The signal of colorimetric sensors is intuitive, does not require processing, and is straightforward to read. The scattering method, which mainly utilizes the specific Raman scattering spectrum of a marker absorbed by the microneedle to detect changes in the marker, relies on the extraction capabilities of the microneedles and requires post‐sampling analysis.^[^ ^64^ ^]^ This technique offers high accuracy, however, integrating the sensing module and achieving point‐of‐care applications presents challenges mainly due to the need for large‐scale equipment.

### 2.2 Fabrication Techniques of Microneedles

The deployed manufacturing strategies are critical for the functionality and application field of the microneedles. Researchers have devised a variety of approaches for fabricating and optimizing microneedle structures, which could be classified into three broad categories: formative, subtractive and additive manufacturing strategies (**Table** **1**).^[^ ^65^ ^]^ Choosing the appropriate technique depends on the specific requirements of the application and the desired microneedle characteristics. To enable mass manufacturing of microneedles, factors such as reproducibility, fabrication precision, lower production cost, and time should be addressed. Techniques such as etching have high production costs. Laser ablation and lithography techniques are costly and time‐consuming. To overcome these limitations, 3D printing, and two‐photon polymerization techniques offer cost‐effective fabrication with high precision and accuracy. Modifications and optimizations of printing parameters within additive manufacturing processes can significantly reduce production time.

**Table 1:** Comparison of different microneedle fabrication methods.

Methods	Materials	Main advantage	Main Limitation	Refs.
Formative Manufacturing Techniques				
Hot embossing	Polymer, hydrogel	Simplicity, ease of implementation, high resolution	Limited precision, time consuming	[66, 67]
Micro‐molding	Polymer, hydrogel	Simplicity, cost‐effectiveness, suitable for complex designs	Design inflexibility due to mold restriction	[51, 68, 69]
Injection‐molding	Inorganic, polymer, hydrogel	Large scale production	Inability to preload detecting materials	[68]
Thermal drawing lithography	Polymer	Direct fabrication of complex 3D structures	Simultaneous control of temperature and motion	[70, 71]
Magnetorheological lithography	Metal, inorganic	Increased morphological controllability, mold‐free fabrication	Dependency on the properties of the fluid, limited applicability	[72, 73]
Subtractive Manufacturing Techniques				
Laser drilling	Metal, polymer	High manufacturing efficiency	Low manufacturing precision, elevated cost	[74]
Deep reactive ion etching (DRIE)	Metal, inorganic, polymer, hydrogel	Precise control over the shape and dimensions, high manufacturing efficiency	Time and cost‐intensive, cleanroom requirement	[46, 68]
Additive Manufacturing Techniques				
3D printing	Metal, inorganic, polymer, hydrogel	Wide customization, design flexibility	Relatively low resolution, limited material options, reliance on elevated temperature, expensive equipment	[31, 75, 76]
Two‐photon polymerization	Polymer	Cost‐effective fabrication	Slow fabrication speed, limited selection of material	[32, 77]
Photolithography	Metal, inorganic, polymer	Controlled precision and replicability	Achieving high aspect ratios, extensive fabrication times, cleanroom requirement	[78]

#### 2.2.1 Formative Manufacturing

In the case of formative manufacturing strategies, the bulk material is processed and shaped accordingly to attain the desired form, while the weight of the materials remains unaltered. Hot embossing is a simple procedure that includes pressing a master mold at high temperature and pressure onto a polymer substrate, thereby replicating the desired needle pattern onto the substrate. The final replication of the needle pattern is determined by the quality of the master mold and the process conditions. It is ideal for low to medium production volumes, but it is crucial to note, that the process might be time‐consuming due to the required heating and cooling cycles.^[^ ^66^, ^67^ ^]^

Micromolding is a process of dispensing a liquid or molten into a mold with the intended shape, to create the final structure of a product. Consequently, the samples must be centrifuged or vacuumed to fill the cavity thus guaranteeing a precise needle tip.^[^ ^68^ ^]^ An additional step of curing with different processes such as cooling the metal with air, water or oil or polymer materials using UV light, can result in tuning the properties of the microneedles. Casting was recently used to develop sodium chondroitin sulfate based solid microneedles suitable for ISF extraction.^[^ ^51^, ^69^ ^]^ Its primary application is in producing solid microneedle arrays, and the surface structure of the microneedles depends on the mold's precision.

Injection molding, similarly, is accomplished by injecting components into a mold and then allowing them to cool and solidify. Since the procedure necessitates intense heating, the molds utilized should be made of thermoresistant materials such as stainless steel or copper‐tungsten substrates and MNs with preloaded detecting materials cannot be manufactured by this technique.^[^ ^68^ ^]^

Thermal‐drawing lithography allows for the direct fabrication of three‐dimensional structures from two‐dimensional thermoplastic and viscous materials.^[^ ^70^ ^]^ A molten polymer is coated onto a baseplate and then brought into contact with drawing pillars, which eventually separate, allowing the polymers to expand and create needle‐shaped structures. The requirement of simultaneous temperature and motion control limits the universal applicability and adoption of this technique, nevertheless it was deployed to fabricate polymer‐based porous microneedles using poly(lactic‐co‐glycolic acid).^[^ ^71^ ^]^

Magnetorheological lithography has been proposed to successfully and mold‐free construct microneedles directly from a droplet of curable magnetorheological fluid in an external magnetic field.^[^ ^72^ ^]^ The process is comparable to thermal‐drawing lithography, but it contributes significantly to reducing manufacturing complexity and increasing morphological controllability; the height of the needles is determined by the volume of the droplet and magnetic field strength, which also determines their sharpness. A flexible array of microneedles was created for transdermal drug administration displaying outstanding penetrating capabilities.^[^ ^73^ ^]^

#### 2.2.2 Subtractive Manufacturing

Microneedles are fabricated using subtractive manufacturing techniques, which involve selectively carving or milling microstructures from substrates. This process effectively removes material, resulting in lighter microneedles compared to the original substrate. Metal materials are frequently subjected to laser drilling. A laser beam's special features of monochromaticity and coherence allow it to focus its power to efficiently vaporise, resulting in the localised removal of material to form microneedles or microholes used in numerous applications.^[^ ^74^ ^]^ Despite high manufacturing efficiency, it can be challenging to fabricate microneedle structures with high precision using standard nanosecond lasers.

In deep reactive ion etching (DRIE), a substrate, usually a silicon wafer, is prepared with a photoresist layer and masked to define the desired pattern. The substrate is then placed in an etching chamber where plasma is generated. The plasma selectively removes material through a combination of physical sputtering and chemical etching.^[^ ^68^ ^]^ DRIE enables the creation of MNs with deeply penetrated and steep‐sided holes and cavities, thanks to highly anisotropic etching processes which can be further enhanced by the formation of passivation layers on the sidewalls. In 1998, the fabrication of the first microneedles for transdermal drug delivery was achieved using DRIE.^[^ ^46^ ^]^ However, DRIE is a time‐consuming process requiring complex and expensive equipment, as well as cleanroom facilities with special treatments.^[^ ^68^ ^]^ Another approach is wet chemical etching, where a mask is utilized to define the desired pattern, but liquid chemicals selectively remove sections of the base substrate to create the microneedles. Needles with uniform thickness are produced, but it cannot achieve pyramidal, tapered, or sharp‐tipped microneedles.^[^ ^68^ ^]^

These micromachining techniques provide design flexibility and customization for various applications, including transdermal drug delivery and biosensing. However, they come with cost considerations due to the need for specialized equipment and skilled operators.

#### 2.2.3 Additive Manufacturing

The most prevalent form of additive manufacturing for microneedles’ fabrication is 3D printing. primarily using light‐cured polymer resins or filaments.^[^ ^31^ ^]^ This approach offers a wide range of design possibilities, for instance, 3D printing has been leveraged in the development of an integrated microneedle biosensing device for continuous monitoring of diabetes.^[^ ^75^ ^]^ The current limitation lies in the relatively low resolution (<50 µm) of commonly available 3D printers, resulting in microneedles with rough surfaces, large radius of base and tip, as well as small aspect ratios.^[^ ^31^ ^]^ The roughness poses challenges in terms of modification and impacts their insertion into the skin, potentially affecting tissue interaction and sensing capabilities.^[^ ^76^ ^]^

Two‐photon polymerization is a versatile and precise additive manufacturing process since it allows for the fabrication of microneedle arrays with exceptional flexibility and high spatial resolution.^[^ ^32^ ^]^ The technique enables the direct creation of complex 3D prototypes from computer‐aided design models, eliminating the need for post‐processing. It offers cost‐effective fabrication of microneedle master templates without requiring harsh chemical treatments being suitable for the fabrication of polymer microneedles for transdermal drug delivery, as reported by Pillai et. al.^[^ ^77^ ^]^ The requirement for precise focusing of laser beams to induce polymerization, resulting in a lengthy fabrication process.

In photolithography microneedle structures are built up layer by layer using light‐sensitive materials and selective exposure to light.^[^ ^78^ ^]^ The process begins with a substrate coated with a photosensitive material, and through a series of steps including photoresist application, exposure, development, and etching, the desired microneedle structures are formed. This additive approach allows for precise control over the shape, size, and arrangement of the microneedles.^[^ ^78^ ^]^ Photolithography and etching excel in achieving close replicas of the intended design; however, they face limitations in creating microneedles with high aspect ratios due to the constraints imposed by lithography substrate projection.^[^ ^68^ ^]^

### 2.3 Biological Matrices Accessed by Microneedles

Microneedles, with their ability to penetrate the biological layers, offer a means to directly access interstitial fluid, cells and tissues to extract valuable physiological information.

#### 2.3.1 Blood

Blood is a vital bodily fluid in humans, responsible for transporting nutrients to tissues and organs while eliminating metabolic waste products from cells. Standard blood tests provide essential biochemical information crucial for diagnosing various diseases and assessing treatment effectiveness. There's a demand for easy, safe, and rapid blood extraction systems for POC sensor‐based diagnosis, and microneedles have gained significant attention as an innovative transdermal blood collection tool.^[^ ^79^ ^]^ Distinct example is the novel lab‐on‐a‐carbohydrate‐microneedle biodevice, which integrates localized surface plasmon resonance (LSPR) technology with microneedles for direct biomarker detection in finger‐prick blood, like cystatin C.^[^ ^56^ ^]^ Also, the one‐touch‐activated blood multidiagnostic system (OBMS) integrates a microneedle and paper‐based sensor to automate blood collection, serum separation, and detection for in vivo glucose and cholesterol diagnosis, enhancing user‐friendly blood diagnostics in healthcare.^[^ ^80^ ^]^

#### 2.3.2 Interstitial Fluid

Microneedle arrays have emerged as a promising technology for real‐time biomarker detection in direct contact with ISF, which constitutes a rich source of biomarkers. Microneedle arrays are capable of easily piercing the SC for rapid and efficient ISF extraction. ISF is generated as a result of blood capillary filtration and surrounds cells and tissues throughout the body. ISF transports nutrients and waste materials between capillaries and cells, as well as signaling molecules, antigens and cytokines to lymph nodes for immunological control.^[^ ^81^ ^]^ In comparison to other biological fluids, ISF is the most comparable to blood in terms of composition, biomarker concentration and temporal patterns.^[^ ^7^ ^]^ Additionally, ISF offers several benefits over blood: it does not clot as a result of the unbiased availability of coagulation factors, showing the potential for continuous biomarker monitoring.^[^ ^82^ ^]^ ISF contains essentially no blood cells and ≈25% protein, providing simpler biological samples.^[^ ^55^ ^]^ ISF can offer local information on the status of the tissue without the necessity for a biopsy due to the contact with surrounding cells.^[^ ^83^ ^]^ Numerous recent metabolomic, proteomic, and transcriptome investigations have demonstrated that ISF contains some distinct biomarkers, some of which are present at amounts greater than those seen in blood.^[^ ^83^, ^84^, ^85^ ^]^ It is worth noting that more than 90% of proteins are shared between the blood and ISF, as revealed from a proteomic analysis sampled by microneedles.^[^ ^8^ ^]^ Also, ≈83% of the proteins present in serum can be found in ISF, but ≈50% of ISF proteins are exclusive to it, implying the potential detection of both unique and common biomarkers in ISF.^[^ ^86^ ^]^ It's worth noting that lymphatic fluid originates from ISF, carrying waste products and undergoing filtration in lymph nodes to maintain tissue health while playing a crucial role in immune system. While microneedles have been employed as drug carriers to access the lymphatic system, they have not been utilized for sensing applications as of yet.^[^ ^48^ ^]^

#### 2.3.3 Cells

Microneedles hold great potential for cell‐level detection of biomarkers, surpassing the limitations of fluorescence techniques. Accurate and rapid detection of biomarkers at the cellular level plays a crucial role in disease analysis and clinical diagnosis. However, current methods for cell‐level detection, such as fluorescence labeling techniques, have limitations. They require expensive instruments, and fluorescent agents, and involve time‐consuming fluorescent labeling. Moreover, issues like photobleaching and cytotoxicity of fluorescent dyes may arise. Using microneedles inserted into or around cells to detect various markers at the cellular level would be a promising approach. Zhou et al. developed a MoS_2_/Pt microneedle for real‐time monitoring of H_2_O_2_ released from Hela cells.^[^ ^87^ ^]^ Lee et al. developed an AuZnOx‐based glucose sensor with a detection limit of 1.2 (± 0.026) µM for glucose, which can be used for glucose detection in cells.^[^ ^88^ ^]^ Researchers utilized solid alginate‐coated poly(L‐lactide) microneedles to recover leukocytes by sampling ISF and viable cells.^[^ ^89^ ^]^ They also enriched antigen‐specific memory cells by incorporating antigen‐ and adjuvant‐carrying nanoparticles into the sampling layer using stimulatory sampling microneedles. This approach could be useful for monitoring important cell populations in vaccination, infectious diseases, and autoimmune disorders.

#### 2.3.4 Tissues

Endoscopy is the most often used and successful diagnostic procedure for internal organs. It is widely utilized for cancer prevention, early detection, and diagnosis.^[^ ^90^ ^]^ However, it may detect only microscopic pathological characteristics and frequently necessitates biopsy sampling of suspected lesions for subsequent histological testing of malignancy, which can be quite unpleasant for the patient. Covering the endoscope surface with microneedles to detect biomarkers is one way to address this issue. A microneedle sensor functionalized with hemin molecules was fabricated at the end of the endoscope for endoscopic microscopy imaging and colon cancer biosensing.^[^ ^91^ ^]^ The obtained microneedle sensor with white light endoscopy can be used in vivo to not only identify polyp areas in the colon, but also to detect enhanced cancer‐specific NO generation. This dual diagnostic approach presented a powerful tool for accurate and swift cancer diagnosis. Microneedles with access to inner tissues can be further explored to replace biopsies for local, precise and minimally invasive tissue diagnosis.

## 3 Construction of Microneedles for Sensors and Diagnosis

Microneedles, as an ideal platform for precise monitoring of biomarkers under the skin, are required to ensure effective skin penetration and reliable needle‐tissue interactions. To achieve painless transdermal sensing, microneedles need to possess appropriate lengths to pierce the epidermis and narrow shape to avoid stimulating the dermal nerves. The irregular surface and inherent elasticity of the skin should be considered for consistent and sufficient insertion. Thus, it is vital to fabricate mechanically strong microneedles to penetrate the dermis without breaking or bending during insertion and daily use. Several studies have determined Young's moduli of SC and essential epidermis, yielding results of 1–1000 Mpa and 2–20 MPa, respectively. Thanks to the great effort in microneedle techniques, the main factors affecting the microneedle performance have been identified as the type of materials and the geometrical parameters (the height, tip radius, base diameter, spacing tip to tip and needle density).^[^ ^92^ ^]^ The ideal microneedles should have a small tip radius to facilitate insertion; and sufficient Young's modulus to avoid fracture. The force required for insertion can be reduced by designing the geometry of the microneedle; the same as the length of the microneedle designed for the target body fluid.

The materials for the fabrication of microneedles must satisfy both good biocompatibility and good mechanical properties. The microneedle‐induced skin contamination such as the breakage of the tips, cytotoxicity, and inflammatory response should be avoided to ensure insertion safety. On this basis, metals, inorganic materials, polymers and hydrogels have become the four main materials to construct microneedle sensors, as shown in **Table** **2**. Generally, these microneedle sensors have the shape of a tapered sharp tip with a height of 250–4000 µm, a base diameter of 50–800 µm, and a spacing base to base of 110–2000 µm. The geometry of microneedles provides the penetration through the epidermis with a thickness of ≈76.9–267.4 µm and the insertion on the dermis with a thickness of 2115–5888 µm.^[^ ^93^ ^]^ Inorganic substances such as metals and silicon generally have higher Young's modulus than polymers, so their aspect ratios of diameter to height are generally smaller, and the tips can be made very sharp (the tip diameter is generally <10 µm). In addition, due to their good electrical properties, the main detection method of microneedles made of these materials is electrochemical detection. The mechanical strength of polymers and hydrogels is relatively weak, so they generally have a large aspect ratio to ensure insertion without risk of damage. Thanks to their structure diversity, multiple functionalization and simple fabrication, such microneedles are applicable for both electrochemical and optical sensing.

**Table 2:** Summary of recent progress in microneedle sensors.

Microneedle material	Type	Height (µm)	Base diameter (µm)	Spacing base to base (µm)	Detection method	Analytes	Ref.
Stainless steel	Hollow	4000	464	/	Potentiometry	Na^+^, K^+^ ion	[94]
Stainless steel	Solid	650	50	150	Surface‐enhanced Raman spectroscopy	Rhodamine 6G	[95]
Stainless steel	Solid	2200	400	/	Amperometry	Glucose	[39]
Stainless steel	Solid	800	225	250	Amperometry	Glucose	[40]
Stainless steel coated by carbon nanotubes	Solid	1000	450	/	Potentiometry	K^+^ ion	[96]
Stainless steel coated by carbon nanotubes	Solid	500	400	/	Potentiometry	pH	[97]
Steel coated by Au layer and PEDOT layer	Solid	680	440	600	Potentiometry	Ca^2+^, Na^+^, K^+^ ions	[98]
Au‐coated silicon	Solid	250	50	110	Amperometry	Glucose	[99]
Au‐coated silicon	Solid	250	50	110	Amperometry	Epidermal growth factor receptor 2	[24]
Au‐coated silicon	Solid	600	200	/	Potentiometry	Vascular endothelial growth factor	[100]
Epoxy siloxane/PDMS	Solid	900	/	/	Potentiometry	pH	[101]
Au‐coated PDMS	Solid	250	50	110	Potentiometry	pH	[102]
Chitosan coated PDMS	Solid	600	300	300	Amperometry	Cell‐free DNA	[103]
Carbon paste in acrylate‐polymer material	Filled‐up hollow	700	800	1000	Amperometry	Opioid and nerve agents	[104]
Carbon paste in 3D‐printed resin	Filled‐up hollow	/	500	1000	Amperometry	Apomorphine	[105]
Carbon paste in acrylate‐polymer material	Filled‐up hollow	800	425	/	Amperometry	Tyrosinase	[25]
Carbon paste in acrylate‐polymer material	Filled‐up hollow	800	500	1000	Amperometry	*β*‐Hydroxybutyrate	[106]
Carbon nanotube/ Poly(lactic acid)	Solid	870	250	1000	Amperometry	Ascorbic acid	[107]
Carbon‐coated polyethylene terephthalate (PET)	Solid	1500	700	/	Amperometry	Glucose	[108]
Gold/silver‐coated polycarbonate	Solid	1000	600	1000	Amperometry	Lactate	[109]
Aptamer‐decorated gold‐coated PMMA	Solid	2264	170	/	Square‐wave voltammetry (SWV)	Tobramycin	[110]
Multilayer coated PMMA	Solid	850	250	/	Amperometry	Lactate, glucose, alcohol	[35]
Polyether ether ketone (PEEK)	Hollow	1000	750	2000	Amperometry	Glucose	[111]
Gold‐coated polystyrene	Solid	1000	750	/	Amperometry	Na^+^ ion	[112]
Polystyrene	Solid	600	300	600	Fluorescence	Protein biomarkers	[37]
Pt and Ag wires in liquid crystal polymer (LCP)	Hollow	800	390	/	Amperometry	Alcohol	[113]
Pt and Ag wires in silk/D‐sorbitol	Hollow	800	400	/	Amperometry	Glucose	[114]
Aptamer‐decorated ETPTA polymer	Porous	950	430	/	Fluorescence	Endotoxin	[115]
Poly(glycidyl methacrylate) and PEG	Porous	600	400	/	Amperometry	Glucose	[40]
SiO_2_ nanoparticles in PEGDA/PEG hydrogel	Hydrogel	800	150	/	Photonic crystal	Inflammatory cytokines	[116]
Hyaluronic acid (HA)	Solid	1344	565	/	Colorimetry	pH, glucose, uric acid, temperature	[117]
SiO_2_ nanoparticles in PEGDA/PAAm hydrogel	Hydrogel	900	400	800	Photonic crystal	Glucose	[118]
Au nanoparticles in PEGDA hydrogel	Hollow Hydrogel	1000	750	/	Plasmonic	Streptavidin	[34]
Methacrylated hyaluronic acid (MeHA) hydrogel	Hydrogel	900	280	/	Amperometry	Glucose	[119]
MeHA hydrogel	Hydrogel	502	323	/	Conductometry	Dopamine	[120]
MeHA/HA hydrogel	Hydrogel	600	/	700	Potentiometry	Ca^2+^, Na^+^, K^+^, H^+^ ions	[121]
Aptamer‐bonded MeHA hydrogel	Hydrogel	850	250	500	Fluorescence	Glucose, ATP, L‐tyrosinamide, and thrombin	[122]
Poly(vinyl alcohol) (PVA)/ chitosan hydrogel	Hydrogel	1266	500	/	Colorimetry	Glucose, chlorine, lactate, BSA	[123]
Ag nanoparticles in HA/PVA hydrogel	Hydrogel	880	470	1000	Surface‐enhanced Raman spectroscopy	Thiram and thiabendazole	[124]
Poly(L‐lactide) coated by Ca2^+^‐alginate matrix	Hydrogel	550	250	340‐400	Fluorescence	miRNA	[38]
Carbon quantum dots in methacrylated gelatin and MeHA hydrogel	Hydrogel	650 ± 20	270 ± 10	600	Fluorescence	miRNA and Cu^2+^ ion	[125]

### 3.1 Metal Microneedle Sensors

Metals and their alloys are embedded with good malleability, easy manufacturing, high conductivity and high mechanical strength, while presenting the risk of immune and inflammatory responses to biological tissues compared to silicon and polymers. Moreover, the use of metal microneedles will cause bio‐hazardous needle waste. Stainless steel was the first metal used and some other metals including titanium, tantalum and nickel have been used in manufacturing microneedles. The Young's moduli of the metal and metal alloys range from 100 to 200 GPa.^[^ ^126^ ^]^ Metals can be used to construct hollow microneedles and solid microneedles with or without a layer of coating, such as the Au coating layer to improve conductivity and biocompatibility.

**Figure 2:**
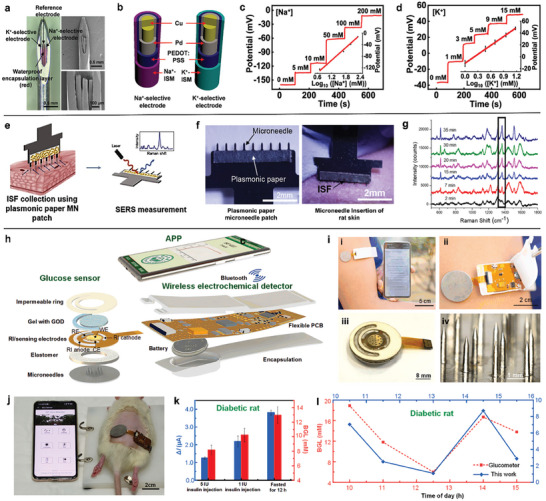
Metal‐based microneedle sensors. a) Optical and scanning electron microscope (SEM) images of microneedle sensing system for Na^+^ and K^+^ detection. b) Schematic illustration of functional layers of the ion‐selective electrodes. c and d) Electrochemical response to different concentrations of Na^+^ and K^+^ ions in artificial ISF. Adapted with permission from 2021 American Chemical Society. e) Simple plasmonic paper‐integrated microneedle for on‐patch SERS detection. f) Image of the microneedle patch and its insertion in rat skin. g) On‐patch SERS measurements of R6G on collected ISF samples. Adapted with permission from 2019 American Chemical Society. h) Schematic illustration of the smartphone‐based glucose electrochemical detection platform (GEDP) integrated with microneedle glucose sensor. i) Photograph of i, GEDP with smartphone readout; ii, microneedle glucose sensor integrated with wireless electrochemical detector; iii, touch‐actuated glucose sensor; iv, solid steel microneedle. j) Photo of the GEDP on a diabetic rat for in vivo glucose detection. k) The comparison between the GEDP and glucometer for three diabetic rat groups. l) The dynamic monitoring of glucose changes for the diabetic rat. Adapted with permission from 2022 Elsevier B.V.

A microneedle‐based potentiometric sensing system was developed for the multiplexed electrolyte monitoring of Na^+^ and K^+^ in artificial ISF and chicken skin model.^[^ ^94^ ^]^ As shown in **Figure** **2**a, a stainless‐steel hollow microneedle was integrated with both sodium ion‐selective electrode (91.2 µm with 7.12% deviation) and potassium ion‐selective electrode (89.3 µm with 5.09% deviation), which are coated with ion‐selective membranes and isolated by the waterproof encapsulation layer at the tip end. The schematics in Figure 2b further display the multi‐layer modification of the sensing electrodes, enabling potentiometric sensing with Ag/AgCl reference electrode. The sensitivity of the Na^+^ sensor at 56.08 mV decade^−1^ and that of the K^+^ sensor at 50.03 mV/decade were evaluated in artificial ISF (Figure 2c,d). More results showed that the potentiometric sensor exhibited a rapid response, excellent storage stability, high reversibility, and negligible potential interference in ISF. Further explorations to improve the biocompatibility and combine it with mobile readout are suggested before conducting in vivo studies.

In addition to the incorporation of sensors inside hollow microneedles, a plasmonic paper‐integrated steel microneedle for on‐patch surface‐enhanced Raman scattering (SERS) was designed (Figure 2e).^[^ ^95^ ^]^ This microneedle sensor can extract ISF on plasmonic paper through a row of 9 microneedles with a length of 650 µm (Figure 2f). This on‐patch sensor realized a direct in situ analysis of rhodamine 6G (R6G) changes on plasma paper, which does not require elution of analytes and requires no additional steps compared to traditional paper‐based microneedles. SERS spectroscopy demonstrated that the characteristic peak of R6G was significantly enhanced over time after inhalation of ISF (Figure 2g). This study shows that microneedles can integrate with plasmonic paper for direct collection of the sample fluid and on‐patch SERS detection of molecules, which enables a low‐cost and portable SERS‐based analytical device with the development of hand‐held Raman spectrometers.

Further exploration of stainless steel microneedles in a wearable analytical device has been developed.^[^ ^39^ ^]^ The microneedle array was integrated with a reverse iontophoresis (RI) unit and a glucose electrochemical sensing unit as the touch‐actuated glucose biosensor, which was assembled with a wireless electrochemical detector and an Android‐based smartphone application (Figure 2h,i). Glucose was transferred to the agarose gel of RI cathode driven by the electroosmotic flow of ISF. The integration of microneedle array with RI fastened the glucose extraction through transient microchannels by 1.6 times than RI itself. The sensing unit was composed of glucose oxidase‐containing hydrogel and electrode system for glucose quantification in chronoamperometry mode. Further in vivo glucose detection was conducted with comparison to commerically available blood glucometer, which showed the high correlation in glucose levels for three healthy and diabetic rat groups (Figure 2j,k). The dynamic glucose changes were monitored in both healthy and diabetic rats, which exhibited similar trend with regard to a time lag for half hour due to glucose diffusion and operation time (Figure 2l). The microneedle sampling strategy improves the effeciency of RI extraction, paving the way for the dynamic monitoring of ISF biomarkers by smartphone‐assisted wearable electrochemical sensors.

### 3.2 Inorganic Microneedle Sensors

Several types of inorganic and carbon materials have been explored as microneedle sensors. Silicon has been used as the exclusive material at the early stage of microneedle development, providing a good tip sharpness but a brittle and easily damaged property. The Young's modulus of silicon ranges from 130 to 180 GPa,^[^ ^126^ ^]^ which is sufficient to withstand the force required to pierce the skin. Ceramic materials and silica glass are rarely explored in microneedle sensors although they have been used in drug delivery for several decades, possibly due to their brittle properties. Recently, siloxane such as polydimethylsiloxane (PDMS), are exploited to construct flexible and large‐area microneedles with electrode or polymer coating for biosensing applications. Except these inorganic materials, carbon materials like carbon paste and graphite have been integrated for microneedle electrochemical sensing due to their excellent conductivity, flexibility and ease of fabrication.^[^ ^127^ ^]^

**Figure 3:**
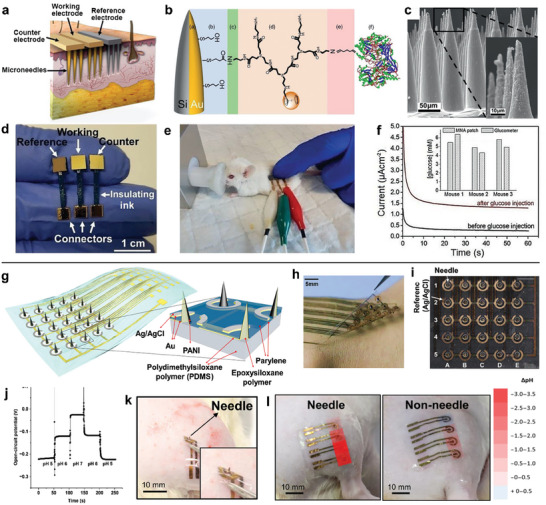
Silicon‐based microneedle sensors. a) Schematic of the three‐electrode microneedle patch penetrating the epidermis and superficial dermis. b) Schematic of the surface modification of the working electrode. c) SEM images of the obtained microneedle array. d and e) The three‐electrode microneedle system used for transdermal monitoring of glucose in mice. f) Amperometric signal of the patch on 3 mice skin recorded before and after glucose injection. Adapted with permission from 2022 Wiley‐VCH. g) Structural schematic of the conformable microneedles on a hybrid substrate for pH sensing. h) Image of 5×5 microneedle sensor array. i) Microscope image of 5×5 microneedle sensor array. j) Open circuit potential (OCP) time curve of a single microneedle pH sensor. k) Photo of microneedle pH sensor on a rate with vascular blockage model. l) The pH distribution provided by microneedle sensors (left) and non‐needle‐type sensors (right). Adapted with permission from 2021 American Association for the Advancement of Science.

A high‐density silicon microneedle array (≈9500 microneedles cm^−2^) was developed for pain free electrochemical detection of glucose.^[^ ^99^ ^]^ This microneedle system provides a large surface area to connect the percutaneous ISF. The glucose‐sensing patch incorporates a three‐electrode system consisting of a reference, working, and counter electrode in a 3D‐printed scaffold (**Figure** **3**a,d). The silicon microneedle was initially coated with gold and then modified to conjugate dendrimers with a redox mediator and the glucose oxidase (Figure 3b). The SEM images of the dense microneedle array shows a tip diameter of ≈2 µm (Figure 3c). The microneedle sensor shows a selective response to glucose in artificial ISF, with a sensitivity of 0.1622 µA mM^−1^ cm^−2^ and a detection limit of 0.66 mM. After inserting the three‐electrode microneedle into the mouse skin (Figure 3e), the amperometric signals of the microneedle sensor were compared with the commercial blood glucose meter, showing a great correlation with blood glucose levels (Figure 3f). With this successful proof‐of‐concept demonstration, the microneedle sensor can be further explored for the continuous transdermal monitoring and the detection of multiple biomarkers.

A flexible microneedle pH sensor array on a hybrid silicone substrate was developed.^[^ ^101^ ^]^ The microneedle integrated two siloxane‐based polymers with different Young's moduli, including epoxysiloxane (3 GPa) as the strong microneedles and PDMS (5 MPa) as the flexible substrate (Figure 3g). The device was achieved with sufficient skin penetration while maintaining skin fit (Figure 3h). The device shows a small change of 6 kilohms after 1000 times insertion into pig skin and a wide range of bending radii, demonstrating its high mechanical durability and flexibility. Polyaniline and Ag/AgCl were electrochemically deposited on the fenestrations of the microneedles for pH sensing (Figure 3i). Measurements of open circuit potentials on 5 × 5 pH sensor array in buffer solutions with different pH showed the average sensitivity up to 94 mV pH^−1^ (Figure 3j). This conformable microneedle sensor was placed on the thigh of rat with vascular blockage model to monitor pH changes, which shows an immediate pH drop of −1.75 after fastening the tourniquet for 5 min (Figure 3k). Compared to the non‐needle‐type pH sensor, the microneedle sensor offered more sensitive and durable results on dermal pH monitoring (Figure 3l), enabling the timely biochemical mapping for under‐skin wearable devices. By integrating two materials with different Young's moduli, this work opens up new ideas of flexible microneedle arrays for skin penetration over large areas of skin.

**Figure 4:**
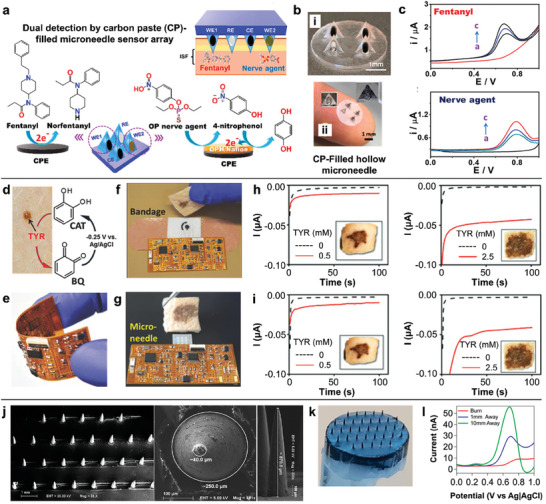
Carbon‐based microneedle sensors. a) Microneedle sensor array for the simultaneous detection of fentanyl and nerve agents. b) Photos of carbon paste‐filled hollow microneedle. c) SMV detection of fentanyl and nerve agents in skin‐mimicking gel. Adapted with permission from 2020 American Chemical Society. d) The on‐body oxidation process from catechol to benzoquinone in the presence of TYR. e) The flexibility of the wireless electronic board. f) The bandage sensor and g) the microneedle sensor integrated with a flexible electronic board. The ev vivo detection of TYR in porcine skin model by h) the bandage sensor and i) the microneedle sensor. Adapted with permission from 2018 Wiley‐VCH. j) SEM images of carbon nanotube‐doped microneedle. k) Microneedle array without damage after 6 turns of skin insertions and removals. l) The DPV responses at progressive distances away from the burn site. Adapted with permission from 2019 American Chemical Society.

Started from the first example of carbon paste‐filled hollow microneedles by J. Wang's group in 2011,^[^ ^128^ ^]^ the carbon paste microneedle sensors with the advantages of low background and convenient surface modification have been widely applied in electrochemical sensing of physiological biomarkers. A wearable microneedle sensor patch with 2 × 2 pyramidal‐shaped hollow microneedles for the continuous monitoring of fentanyl and organophosphate nerve agents was developed (**Figure** **4**a).^[^ ^104^ ^]^ The unique microneedle substrate was fabricated by poly(methyl methacrylate) (PMMA) using the CNC micromachining technique. A close‐view optical image of the microneedle array shows that three hollow microneedles are packaged with modified carbon paste as the working electrodes (WE1 and WE2) and unmodified carbon paste electrodes (CE), while the fourth was filled by Ag/AgCl wire as the reference electrode (RE) (Figure 4b). The simultaneous square wave voltammetric (SWV) analysis of fentanyl and organophosphate nerve agents was realized with high sensitivity (down to 50 nM for fentanyl detection), selectivity and stability. Figure 4c verified the well‐defined voltametric response of both analytes in the skin‐mimicking phantom gel (1% agarose), indicating the promising on‐body monitoring in the future. Such a multiplexed transdermal sensing device, capable of distinguishing between episodes of opioid overdose and nerve agent poisoning, can be very advantageous toward a timely life‐saving medical intervention.

By immobilizing the tyrosinase (TYR) substrate catechol in the carbon‐based working electrodes, a non‐invasive carbon printing bandage sensor and a minimally invasive carbon paste‐filled microneedle sensor were constructed to detect TYR enzyme biomarkers for early skin cancer diagnosis.^[^ ^25^ ^]^ The production of benzoquinone by the oxidation of catechol in the presence of TYR can be measured amperometrically at a potential of −0.25 V (Figure 4d). The bandage and microneedle sensors were incorporated with a flexible electronic board containing potentiostatic circuitry and wireless data transmission for on‐body amperometric detection (Figure 4e). To evaluate the sensing performance of the bandage sensor (Figure 4f) and the microneedle sensor (Figure 4g), porcine skin was dosed with different TYR levels for 24 h, resulting in a skin color change from pink to dark brown. As illustrated in Figure 4h,i, both wearable sensors demonstrated the distinct current changes on the treated pork skin at different TYR concentrations. With the potential multiplexed microneedle sensing, the fully integrated wearable sensors with a flexible wireless reusable electronic board developed in this work implied a promising on‐body melanoma screening.

In addition to the characterization of biomarkers in ISF, the characterization of skin tissue is also an area of interest. A poly(lactic acid)/carbon nanotube composite microneedle array was developed for dermal biosensing.^[^ ^107^ ^]^ The microneedle array sensor with the maximum carbon nanotube loading of 6 wt.% was fabricated by solvent‐cast micromolding (Figure 4j), which enhanced its mechanical stiffness than undoped microneedles. No evidence of damage to the microneedle was identified after 6 repetitive skin insertions and removals (Figure 4k). The doping of carbon nanotubes can also reduce the overpotential required for ascorbic acid oxidation, allowing the microneedles to detect ascorbic acid levels with a limit of detection of 180 µM by differential pulse voltammetry (DPV). The sensitive detection of this oxidative species can be applied in the detection of skin burns, and the electrochemical results show that the peak intensity decreases significantly in the burned skin (Figure 4l). The carbon nanotube‐based microneedle offers a rapid and dynamic electrochemical sensing of skin burns, which can be further explored for the monitoring of burn wound recovery.

### 3.3 Polymer Microneedle Sensors

Polymer microneedles have been widely developed because of their good biocompatibility, low cost, easy to mold, and potential for mass production. A broad range of polymer materials including PMMA, polystyrene (PS), polylactic acid (PLA), polycarbonate (PC), polyethylene (PE), polyurethane (PU), polyether ether ketone (PEEK), and cycloolefin polymers have been exploited in microneedle fabrication. With a range of Young's modulus from 0.7 to 4.5 GPa,^[^ ^126^ ^]^ these materials present sufficient hardness to penetrate tissue barriers such as the skin and cornea. Some soft polymer microneedles are prone to buckling failure during the tissue insertion process, which need be improved by a rational material design. It was suggested to construct microneedles with an aspect ratio of diameter to height over 1:12 and Young's modulus above 3 GPa to reduce buckling.^[^ ^129^ ^]^ Moreover, most of these polymer materials are biocompatible and/or biodegradable, which avoids the production of bio‐hazardous tip wastes. Low‐cost fabrication techniques like micro‐molding or hot embossing are beneficial for the mass manufacture of biomedical products.

**Figure 5:**
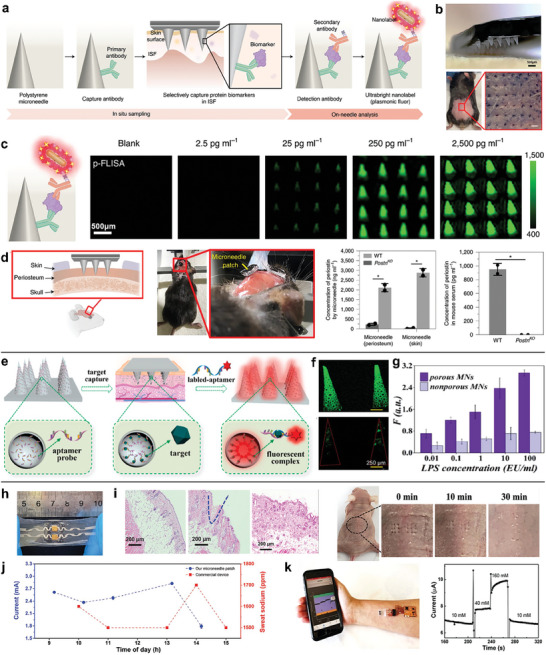
Polymer‐based microneedle sensors. a) The construction of ultrasensitive fluorescent microneedle sensors for in situ sampling and on‐needle detection of protein ISF biomarkers. b) Optical image of the magnetic backing layer microneedle patch and its penetration on mouse skin. c) Fluorescence intensity maps of mouse IL‐6 at various analyte concentrations enhanced by plasmonic effect. d) Microneedle measurements of endogenous biomarkers in mouse calvarial periosteum. Adapted with the permission from 2021 Springer Nature. e) Schematic illustration of the aptamer‐decorated porous microneedle sensors. f) The confocal images of porous and nonporous microneedles immobilized by fluorescent aptamers. g) The relative fluorescent intensity of microneedles after the capture of ISF biomarkers. Adapted with permission from 2021 Elsevier B.V. h) A flexible microneedle‐EGFET biosensor patch for Na^+^ detection. i) H&E staining images and photos after microneedle insertion into mice and recovery of pinholes within 30 mins. j) The long‐term on‐body Na^+^ detection of microneedle sensor compared to a commercial sweat sensor. k) Wireless Na^+^ detection at different concentrations assisted by the smartphone. Adapted with permission from 2022 Wiley‐VCH.

A microneedle‐based ultrasensitive fluorescent sensor has been exploited for various ISF protein biomarkers with limit of‐detection almost 800‐fold lower than conventional fluorophores.^[^ ^37^ ^]^ Through in vivo sampling and in vitro on‐needle analysis, the microneedles functionalized by the specific antibody can selectively capture the corresponding biomarkers in ISF and then quantified by nanogold‐enhanced plasmonic fluor‐linked immunosorbent assay (**Figure** **5**a). The bilayered microneedle patch, which was prepared by micromolding of PS with a backing layer of PS and magnetic (Fe_3_O_4_) nanoparticles. The mouse ventral skin penetrated by the microneedle arrays was measured with a depth of 140‒170 µm and recovery time of 15 min (Figure 5b). The developed plasmonic‐enhanced fluoroimmunoassay on microneedle patch were proved as a simple, quantitative and highly sensitive analytical tool to protein biomarkers (Figure 5c). The minimally invasive detection assured an efficient sampling of the calvarial periosteum without the potential death by conventional invasive procedures (Figure 5d). To explore the potential capabilities in biomedical research, the microneedle patch functionalized with varied antibodies has been applied to monitor long‐term inflammatory immune responses, assess vaccine efficiency, and determine local changes of protein content in target tissues.

Except for the incorporation of commonly used antibodies as recognition sites, aptamer‐decorated porous microneedle sensors have been developed for the advantages of aptamers including high detection specificity, easy modification and thermal stability (Figure 5e).^[^ ^115^ ^]^ The porous microneedles were fabricated by UV‐curable ethoxylated trimethylolpropane triacrylate (ETPTA) mixed with glass microspheres. The aptamer probes were immobilized and bridged by a coating of polydophamine. The fluorescent intensity of porous microneedles after the aptamer immobilization was greatly enhanced than that of nonporous microneedles (Figure 5f). To monitor infectious diseases triggered by gram negative bacteria, the aptamer microneedle sensors were applied for a rapid and highly sensitive detection of lipopolysaccharide (LPS) (Figure 5g).

Apart from the fluorescent sensors, a PS‐based stretchable and skin‐conformal microneedle extended gate transistor was developed for real‐time, fast‐responsive and sensitive detection of Na^+^ ion (Figure 5h).^[^ ^112^ ^]^ As shown in Figure 5i, the dermal layer of mice skin tissue was reached by the insertion of microneedle and the micropores were fully recovered 30 min after the removal of microneedle, indicating the effective access to ISF and no skin damage of PS microneedle. The rapid recovery and resealing of the skin is crucial to prevent the invasion of pathogenic microorganisms and reduce the risk of inflammatory or toxic response. After the validation of sensing performance and biocompatibility, the on‐body ISF measurements of a healthy individual were performed with comparison to sweat measurements by a commercial Na^+^ meter (Figure 5j). The Na^+^ concentration fluctuation showed a similar tendency while the sweat measures had a time delay of ≈40 min. To promote remote healthcare monitoring, the wearable microneedle patch was integrated with a wireless Bluetooth transmitter and combined with a smartphone readout (Figure 5k). The fast, real‐time and long‐term monitoring of Na^+^ levels were realized by the integration of advanced data communication tools and microneedle platform.

**Figure 6:**
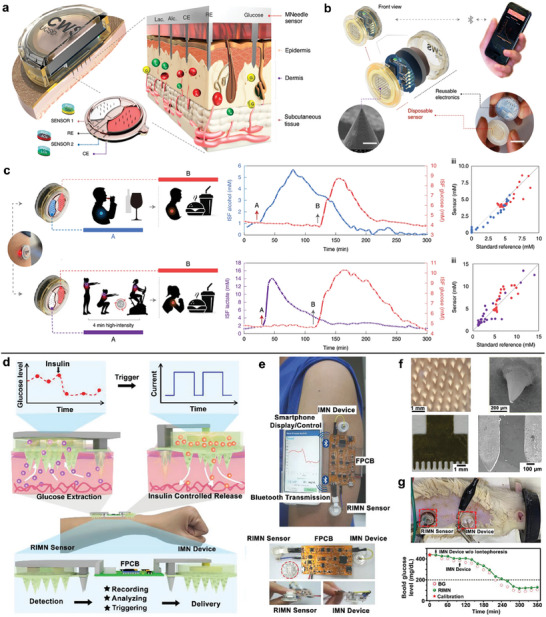
Polymer‐based microneedle sensors with system integration. a) The integrated microneedle patch for continuous and multiplexed sensing. b) The exploded view of device components as reusable electronics and a replaceable sensor array. c) The on‐body performance of dual‐analyte microneedle sensors during wine consumption, meal consumption and high‐tensity workout events, with comparison to the analyte reference measurements in blood or breath. Adapted with permission from 2022 Springer Nature. d) Illustration of the microneedle‐based IWCS for simultaneous diabetic sensing and treatment. e) Images showing the different components of IWCS, which is wirelessly communicating with a smartphone. f) Photos showing the morphology of porous microneedle patch and 2D‐metal microneedle. g) Measurement of dynamic glucose signals upon simultaneous application of the controlled insulin delivery on a diabetic rat model. Adapted with permission from 2021 Wiley‐VCH.

The integration and multiplexing of microneedle sensors with robust analytical performance are a great challenge that hinders their practical application. A fully integrated wearable microneedle sensors for the wireless and continuous monitoring of two metabolites has been developed and validated by the in vivo measurements on the individuals performing daily activities (**Figure** **6**a).^[^ ^35^ ^]^ The device consists of reusable electronics and a disposable microneedle array for cost‐effective fabrication and pain‐free skin penetration. The acquired electrochemical signals can be wirelessly transmitted to the accompanying smartphone app for data capture and visualization (Figure 6b). To develop the selective detection of lactate, alcohol and glucose, the PMMA‐based microneedles were coated by various polymer layers including the respective oxidase enzyme in chitosan. After the validation of single‐analyte sensors, the simultaneous dual‐analyte monitoring such as glucose‐alcohol or lactate‐glucose was measured, which showed a Pearson's r of 0.81‐0.98 with the corresponding reference measurements in blood or breath (Figure 6c). Such a high correlation with gold standard methods illustrated the practicality of wearable sensing technology in real‐world scenarios, which can be further validated in large populations.

Toward the trend of theranostic technologies, a closed‐loop microneedle system (IWCS) has been developed for wearable diagnosis and treatment of diabetes, enabling in situ glucose detection and insulin delivery (Figure 6d).^[^ ^40^ ^]^ The IWCS is composed of three connected components: a reverse iontophoresis‐combined microneedle sensor, a flexible printed circuit board, and a microneedle iontophoresis‐assisted transdermal drug delivery system (Figure 6e). To facilitate the substance exchange, the mesoporous microneedles were fabricated by polymerizing poly(glycidyl methacrylate) (PGMA) and PEG as a porogen (Figure 6f). The 2D‐metal microneedle array was employed as the counter electrode for both iontophoresis and reverse iontophoresis. The incorporation of reverse iontophoresis in microneedle sensor increased the glucose extraction by 452‐folds and the detection sensitivity by 3.8‐folds. Furthermore, the plasma insulin levels of diabetic rats treated by the iontophoretic microneedle device were enhanced to 113.8 ± 9.9, which is almost 2 times the common microneedle. As shown in the in vivo mice measurements, the IWCS system realized the simultaneous glucose monitoring and the treatment of hyperglycemia by insulin administration (Figure 6g). In the above three works, the integration of mobile devices and wearable sensors brings the possibility of remote healthcare monitoring and reduces hospital burdens for medical diagnosis.

### 3.4 Hydrogel Microneedle Sensors

Hydrogel is an emerging microneedle material since its first report in 2012.^[^ ^33^ ^]^ Hydrogel microneedles possess great potential in biomedical applications due to their advantages of excellent biocompatibility, easy modification, and mechanical flexibility. The unique swelling property of hydrogels enables them to uptake ISF as a source of biomarkers or deliver drugs with a high loading capacity and tunable releasing rate. Hydrogel microneedles with relatively weak mechanical strength are mostly utilized in a small penetration depth for minimally invasive and pain‐free diagnosis. Currently, materials including alginate, hyaluronic acid (HA), methacrylated hyaluronic acid (MeHA), poly(ethylene glycol) (PEG), poly(methyl vinyl ether‐co‐maleic anhydride) (PMVE/MA), poly(vinyl alcohol) (PVA), poly(hydroxyethyl methacrylate) (pHEMA), poly(L‐lactide) (PLLA) and silk fibroin^[^ ^130^ ^]^ have been explored to fabricate hydrogel microneedles.

**Figure 7:**
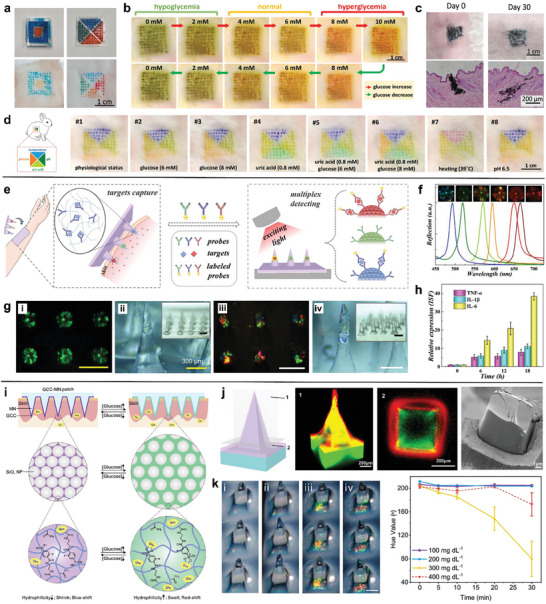
Hydrogel‐based microneedle colorimetric sensors. a) Photos of patterned microneedle patches and skin tattoos injected by microneedle patches. b) Reversible glucose detection using the colorimetric skin tattoo biosensor. c) Photos and H&E slice staining of rabbit's skin before and after inserting microneedle dermal tattoo ink. d) Simultaneous colorimetric detections of four biomarkers in vivo. Adapted with permission from 2021 Wiley‐VCH. e) Schematic illustration of the PhC‐encoded microneedle sensors for the multiplexed detection of inflammatory cytokines. f) The reflection spectra and optical images of different kinds of PhC barcodes. g) Optical images of microneedles loaded by one barcode and three barcodes. h) Relative expression of TNF‐α, IL‐1β and IL‐6 detected by encoded microneedle sensors. Adapted with permission from 2019 Wiley‐VCH. i) Schematic illustration of the glucose‐responsive colloidal crystal‐modified microneedle. j) Confocal microscopy and cross‐sectional SEM images on different sections of microneedle. k) Photos and the averaged hue value of the microneedle sensors treated with glucose solutions in different concentrations. Adapted with permission from 2020 Elsevier B.V.

By constructing a four‐region segmented microneedle patch, a colorimetric skin tattoo biosensor was developed for the multiplexed detection of pH, glucose, uric acid and temperature.^[^ ^117^ ^]^ The fast dissolvable HA‐based microneedles were utilized to encapsulate the colorimetric reagents and release them in dermis after skin insertion (**Figure** **7**a). The hue values of four tattoo sensors administered by the microneedles were measured *ex vivo* on rabbit skin in different conditions. For example, the glucose levels in the conditions of hypoglycemia, normal and hyperglycemia were reversibly and sensitively detected (Figure 7b). The tattoo sensors still clearly existed in the dermis layer after 30 days (Figure 7c), indicating the potential for long‐term monitoring. A microneedle patch with four isolated patterned areas was then prepared for multiple sensing over 4 days, proving its superiority in detecting multiple biomarkers simultaneously (Figure 7d). This work opens up the diagnostic applications of hydrogel microneedles with biocompatibility and rapid biodegradation for long‐term and multiplexed health monitoring.

The ISF multivariate analysis of inflammatory cytokines was developed by the integration of photonic crystal (PhC) barcodes on the tips of microneedles and the subsequent formation of sandwich immunocomplexes (Figure 7e).^[^ ^116^ ^]^ Compared to fluorescent barcodes, PhC barcodes display the unique advantages of narrow reflection peaks and low background interference. PhC barcodes with different structural colors were loaded on the tips of PEG‐PEGDA microneedles, showing distinguishable reflection peaks (Figure 7f). This simple approach provided the loading of one to three barcodes on the sample tip, implying the adjustable and versatile fabrication of encoded microneedles (Figure 7g). By decorating the corresponding antibodies on the surface of barcodes, the microneedle sensors showed accurate, specific and multiplexed detection of TNF‐α, IL‐1β and IL‐6 in a sepsis mice model (Figure 7h). This encoded microneedle platform paves the way for ISF detection in a simple, flexible, and multiplexed manner.

A colloidal crystal‐based colorimetric microneedle sensor was developed for naked‐eye glucose monitoring.^[^ ^118^ ^]^ The microneedle sensor was fabricated by embedding periodic structures of SiO_2_ nanoparticles in a hydrogel matrix, which was photopolymerized by fluorophenyl boronic acid, poly(ethylene glycol) diacrylate and acrylamide (Figure 7i). During hyperglycemia with the increased glucose molecules, the hydrogel swells and the SiO_2_ spacing is expended, leading to a redshift in the reflection spectrum. The 3D confocal microscopy images and SEM images of the microneedles demonstrate the successful coating of the responsive coating on the microneedle array (Figure 7j). By optimizing the hydrogel sensor, a large spectral shift of 127 nm is observed in the physiological glucose levels (100‐400 mg dL^−1^). Such an excellent glucose sensitivity can be easily recognized by naked eyes (Figure 7k). When the glucose concentration was greater than 200 mg dL^−1^ in hyperglycemia conditions, the microneedles could observe a significant red shift over time. This microneedle sensor has been validated for in situ detection of hyperglycemia in the type 1 diabetic mouse model. The hydrogel microneedle reduces inflammation concerns and a platform to tailored for broad biomedical applications.

**Figure 8:**
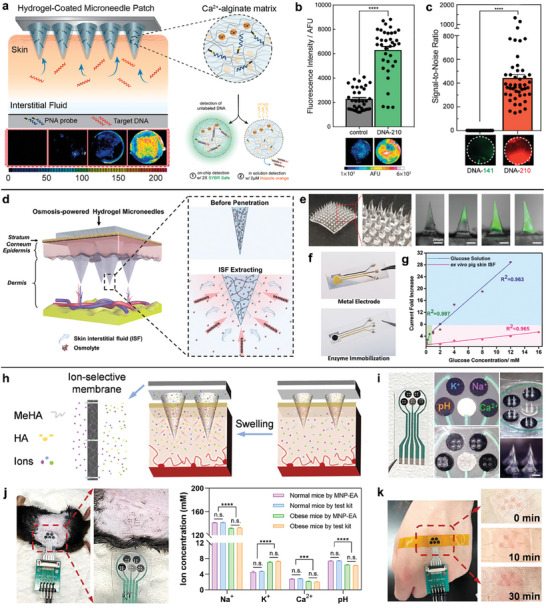
Hydrogel‐based microneedle fluorescent and electrochemical sensors. a) A hydrogel‐coated microneedle platform for the detection of specific circulating nucleic acids. b) On‐chip detections of the DNA target and control samples. c) Selective detection of fluorescent labelled DNA‐210 from the mixed DNA human skin samples. Adapted with permission from 2019 American Chemical Society. d) Osmosis‐powered hydrogel microneedle patch for effective ISF extraction. e) Photos of microneedles after the skin insertion to mouse back and after the ISF extraction from porcine skin containing different concentrations of biomarkers. f) The electrochemical glucose sensor strip to be integrated with the microneedle path. Adapted with permission from 2020 Wiley‐VCH. g) The amperometric response of glucose sensor in vitro and *ex vivo* porcine skin. h) The swellable hydrogel microneedle coupled with ion‐selective membrane for electrochemical sensing. i) Multiplexed ion sensing on the flexible microneedle patch. j) In vivo multiplexed ion detection on normal and obese mice compared to commercial assays. k) Microneedle ion sensing on human hand with holes faded in 30 min. Adapted with permission from 2023 American Chemical Society.

To realize the fluorescent detection of cell‐free nucleic acid biomarkers in ISF, the Ca^2+^‐alginate hydrogel functionalized with peptide nucleic acid (PNA) capture probes were coated on the 7 × 7 mm^2^ PLLA microneedle arrays for sequence‐specific detection with high affinity (**Figure** **8**a).^[^ ^38^ ^]^ The obtained microneedle sensor was characterized by fast sampling (≈6.5 µL in 2 min) and specific miRNA isolating. This versatile platform is capable of both in situ detection of miRNA for on‐chip analysis and UV‐triggered release of miRNA for off‐chip analysis. Both strategies proved the quantitative detection of nM concentrations of nucleic acids. For on‐chip analysis, the bound DNA sampled by microneedles was visualized by the addition of a fluorescent DNA intercalator (Figure 8b). The sequence specificity of this platform was further provided by capturing the target DNA in the ex vivo human skin model incubated by mixed DNA (Figure 8c). Interrogation of clinically informative nucleic acid biomarkers in ISF by microneedle sensors is promising to transform molecular diagnostics.

To facilitate the ISF sampling and rapid electrochemical sensing, an osmosis‐powered microneedle patch was constructed by a highly swelling MeHA hydrogel and an osmolyte (maltose).^[^ ^119^ ^]^ The additional osmotic pressure from the introduction of osmolytes increased the extraction of ISF from the skin to the hydrogel matrix (Figure 8d). The patch with 100 microneedles can extract 7.90 µL of ISF from porcine skin and 3.82 µL from mouse skin in 3 min (Figure 8e). The in situ analysis of glucose can be achieved by directly integrating the electronic glucose sensor on the backing layer of the microneedle patch (Figure 8f). The results show that the sensor exhibits a good linear response in the range of glucose concentrations from 0 to 8 mM in the pig ear skin model (Figure 8g). The addition of osmolytes in hydrogel microneedles proved to be an effective strategy to improve the skin ISF collection, which is beneficial for rapid point‐of‐care diagnosis.

Except the addition of osmolytes, mixing the dissolvable HA in the crosslinked MeHA matrix was explored to improve the ISF extraction (≈6.87 µL needle^−1^ in 5 min) while maintaining sufficient mechanical strength (Figure 8h).^[^ ^121^ ^]^ The optimized MeHA/HA hydrogel microneedle was then coupled with a flexible electrode array for the multiplexed sensing of Ca^2+^, Na^+^, K^+^, and H^+^ ions in skin and plant ISF. With reference to the central Ag/AgCl electrode, the surrounding working electrodes were coated with ion selective membranes, producing an open‐circuit potential proportional to the target ion accumulation (Figure 8i). The in vivo microneedle monitoring of four electrolytes on normal and obese mice were compared with commercial assays, in which similar results confirmed the practical feasibility of the obtained potentiometric sensor (Figure 8j). This microneedle sensor was also successfully applied to monitor electrolyte changes of human hands after excise and tomato plants under salinity stress (Figure 8k). Microneedle sensors are not only critical for point‐of‐care health monitoring, but also offer a cutting‐edge platform for smart agriculture.

## 4 Commercialization and Clinical Trials of Microneedle sensors

While great efforts have been devoted into creating microneedle sensors in the laboratory, the commercially available microneedle devices are still limited in the market. Current commercialization of microneedle sensors has been focused on continuous glucose monitoring (CGM), such as Dexcom G6, Abbott Freestyle Libre 2, and Medtronic Guardian. Instead of finger‐pricking blood sampling, the microneedle devices provide a conformable and real‐time monitoring of glucose for diabetic patients. Despite the commercial success of CGM devices, the high cost and short working time have restricted the widespread use of microneedle‐based systems. Further exploration of low‐cost and large‐scale microneedle manufacturing technologies is promising to promote the practical translation of microneedle sensors.

Several clinical trials are currently underway to assess the safety and effectiveness of microneedle biosensors in diagnosing various diseases and conditions.^[^ ^131^ ^]^ A comprehensive literature search was conducted using keywords “microneedle” and “biosensor” across multiple databases, including the National Center for Biotechnology Information, ClinicalTrials.gov, EU Clinical Trials Register, ISRCTN Registry, World Health Organization International Clinical Trials Registry Platform (ICTRP), Australian New Zealand Clinical Trials Registry (ANZCTR), Chinese Clinical Trial Registry (ChiCTR), Japan Pharmaceutical Information Center Clinical Trials Information (JapicCTI), Korean Clinical Trial Registry (KCT), and Clinical Trials Registry – India (CTRI). The identified trials in **Table** **3** collectively highlight the promise of microneedle‐based sensors in applications ranging from medication adherence monitoring in opioid use disorder treatment to disease progression tracking in conditions like Parkinson's and diabetes. The integration of microneedles with cutting‐edge electrochemical and molecular technologies demonstrates a valuable approach to continuously monitor a wide range of analytes, holding the potential to significantly enhance patient care and treatment management. Moreover, comparative studies conducted within these trials suggest that microneedles may offer safer and more efficient alternatives to traditional monitoring methods. Particularly in pediatric populations, where comfort and ease of use are paramount, microneedles could make healthcare more accessible and less daunting for patients. However, it is crucial to acknowledge that while these trials show considerable promise, further research and development are needed to validate their widespread clinical use. Addressing technical challenges and ensuring the safety and reliability of microneedle‐based biosensors will be essential to fully unlock their potential in improving healthcare outcomes across various medical disciplines.

**Table 3:** Summary of clinical trials deploying microneedle‐based biosensors.

Clinical trial	Analyte sensed	Condition	Subjects	Summary	Ref.
KCT0008534 NCT05922176	RNA	Allergic rhinitis	30	Transcriptomic prognostic evaluation of clinical indicators based on RNA extracted from skin samples before immunotherapy in allergic diseases.	[132]
NCT05546229	Methadone Buprenorphine	Opioid use disorder	11	Detectability assessment of common medications used in opioid use disorder treatment and their respective metabolites in dISF utilizing microneedles.	[133]
NCT05998876	Methadone	Opioid use disorder	45	Detection of methadone in ISF with differential pulse voltammetry integrated on a microneedle electrode array, for methadone adherence continuous monitoring.	[134]
NCT04735627	Levodopa	Parkinson's disease	20	Microneedle electrochemical sensor for real‐time monitoring of levodopa levels in people with Parkinson's disease.	[135]
NCT03847610	Beta‐lactam	Antimicrobial resistance	11	Efficacy evaluation of a microneedle electrochemical biosensor for monitoring benzylpenicillin levels in comparison to microdialysis and blood sampling methods.	[136, 137]
NCT04238611	Lactate	Anaerobic thresholds	15	Validation of a microneedle‐based device for the continuous measurement of lactate during exercise.	[138]
NCT02682056	Glucose	Paediatric diabetes	15	Efficacy comparative study of microneedle patch over lancets or intravenous catheters for monitoring glucose levels among the pediatric diabetic population.	[139]
NCT01908530	Glucose	Type‐1 diabetes	26	Safety and efficiency evaluation of an electrochemical microprobe array incorporating entrapped glucose oxidase for continuous glucose sensing.	[140]

## 5 Conclusion and Future Prospects

The development of microneedle sensors has fundamentally transformed the invasive blood‐centered diagnostics by providing direct and continuous access to ISF. This review provides an overview of recent advances in microneedle sensors across different aspects of materials science for their upcoming translations in POC diagnostics and personalized medicine. A wide range of materials and fabrication methods have been established to construct microneedle sensors with diverse geometries, mechanical flexibility, and biocompatibility. The integration of various sensing technologies, mainly electrochemical and optical sensors, have been realized in multiple forms of “lab‐on‐a‐microneedle” techniques. Microneedles with complex and tailored geometries have been acquired with emerging additive manufacturing techniques like 3D printing and two‐photon polymerization. ISF holds a leading role as the biological matric accessed by microneedles to extract rich physiological and biomarker information in a minimally invasive and painless manner. Microneedle sensors based on metals, inorganic materials, polymers, and hydrogels have been elaborated with advanced materials and bioengineering technologies for continuous, accurate, reliable, and efficient diagnostics. For example, the combination of reverse iontophoresis has enhanced both ISF extraction and sensitivity.^[^ ^39^, ^40^ ^]^ The hybrid or composite materials can strengthen the mechanical flexibility and functional adaptability.^[^ ^101^, ^107^ ^]^ Signal amplification via plasmonic effect can significantly improve optical sensitivity.^[^ ^37^, ^95^ ^]^ By immobilizing biorecognition elements like antibodies, peptides, and aptamers, the detection range has expanded from simple metabolites (e.g., electrolytes, glucose, and lactate) to complex compounds (e.g., hormones, vitamins, proteins, nucleic acids, and drugs). Various strategies have been applied to endow multiplexed sensing capabilities,^[^ ^117^, ^121^, ^123^ ^]^ especially in hydrogel microneedles thanks to their facile on‐needle functional modifications.^[^ ^141^ ^]^ Theragnostic microneedle systems combining biosensors and drug delivery are emerging despite their manufacturing difficulties.^[^ ^40^, ^142^ ^]^ More importantly, the incorporation of microneedle sensors with power supply, wireless communication, data capture and visualization components integrated in a close‐loop system have been realized.^[^ ^35^, ^112^ ^]^ These remarkable achievements in microneedle system integration are expected to promote the clinical transformation of wearable technologies.

Along with the insight into the latest microneedle technologies, the current challenges and further opportunities toward clinical translation are recognized from the viewpoints of biofluid, microneedles, biosensors and POC devices. i) The diffusion of size‐dependent analytes from blood to ISF is not fully understood, thus the effect of ISF sampling through microneedles on analyte detections is uncertain.^[^ ^8^ ^]^ A lack of clinical data on the correlation of biomarker concentrations between ISF and blood impedes the development of effective diagnostic tools. ii) The large‐scale and cost‐effective manufacturing technologies are urgently required to fasten the commercialization and versatile use of microneedle arrays, which is particularly attractive for resource‐limiting settings.^[^ ^143^ ^]^ Future studies should evaluate material and fabrication costs and the feasibility for industrial scale‐up. The impact of different skin elasticity on the microneedle penetration depth and detection accuracy can be reduced by rational geometric design and standard calibration procedures.^[^ ^144^ ^]^ The integration of well‐defined microfluidics in microneedle sensors promises to reduce the required ISF volume to the nanoliter level and enrich multiplex analysis methods.^[^ ^145^ ^]^ iii) Real‐time and in vivo detection of multiple biomarkers toward a specific disorder or disease is highly desired for a cohesive study of physiological and pathological processes. High sensitivity and selectivity can be achieved by the exploration of nanotechnology in biosensing, such as the integration of nanostructures (e.g., gold, carbon, photonic crystals) in hydrogel microneedle.^[^ ^146^ ^]^ While microneedle sensors have been calibrated by various preclinical tests in skin‐mimicking phantom gel, sectioned animal skin and animal models, the need for clinical trials in humans is inevitable to eventually validate the biosafety and practical performance. The time lag and sensor bias due to the blood‐to‐ISF diffusion, in situ interaction with microneedles and even off‐site measurements need be assessed for continuous monitoring.^[^ ^147^ ^]^ Meanwhile, the long‐term stability of microneedle sensors (e.g., days) can be reinforced to avoid frequent replacement for robust and user‐friendly diagnosis. iv) Prototyping self‐powered, wireless and miniaturized POC devices integrated with a conformable microneedle sensor can accelerate practical translation by bridging the gap between academic research and industry.^[^ ^148^ ^]^ High‐resolution smartphone cameras with the ability to capture light signals quickly and accurately facilitate the development of optical biosensing in POC diagnostics.^[^ ^149^ ^]^ The communication and network functions of smartphones also provide solid basis to automate the data transfer, sensor readout and cloud storage. Artificial intelligence techniques empower high‐dimensional big data analytics for next generation healthcare technologies toward personalization, digitization and intellectualization.^[^ ^150^ ^]^ We envision microneedle sensing technology with huge potential to achieve widespread and mature adoption through extensive interdisciplinary efforts, paving the way for revolutionizing patient‐centric healthcare and clinical scenarios with wearable devices.

## Conflict of Interest

The authors declare no conflict of interest.
